# Metastases to the thyroid gland from uncommon primaries: a case series

**DOI:** 10.1210/jcemcr/luag119

**Published:** 2026-05-14

**Authors:** Aabharana Rao, Akhila Lakshmikantha, Swarathika Majumdar, Anirudh Shetty, Nisheena Raghavan, Subramanian Kannan

**Affiliations:** Junior Registrar, Department of General Medicine, Narayana Health, Bangalore, Karnataka 560099, India; Consultant, Department of Histopathology, Narayana Health, Bangalore, Karnataka 560099, India; Consultant, Department of Medical Oncology, Narayana Health, Bangalore, Karnataka 560099, India; Consultant, Department of Endocrinology, Diabetes & Metabolism, Narayana Health, Bangalore, Karnataka 560099, India; Consultant, Department of Histopathology, Narayana Health, Bangalore, Karnataka 560099, India; Consultant, Department of Endocrinology, Diabetes & Metabolism, Narayana Health, Bangalore, Karnataka 560099, India

**Keywords:** thyroid metastasis, fine-needle aspiration cytology, FNAC, immunohistochemistry, IHC, uncommon primary cancers, positron emission computed tomography imaging, PET-CT

## Abstract

Metastasis to the thyroid gland is an uncommon clinical finding that is often mistaken for primary thyroid carcinoma because of overlapping radiologic and cytologic features. We report three patients with thyroid metastases who underwent clinical evaluation, ultrasound, fluorodeoxyglucose positron emission–computed tomography, fine-needle aspiration cytology, and confirmatory histopathology with immunohistochemistry (IHC) to establish the primary source. All three patients presented with thyroid nodules classified as Bethesda VI on cytology with cellular features pointing toward a nonthyroid origin. Cell block preparation and, in selected cases, IHC were essential for distinguishing metastases to the thyroid gland from a primary thyroid carcinoma. The primary tumors identified in this series were a gastric adenocarcinoma with signet ring morphology, a choriocarcinoma, and a leiomyosarcoma. These cases highlight that, although rare, thyroid metastases should be considered in the differential diagnosis of atypical thyroid nodules. In cases where sonographic findings resemble those of primary thyroid cancer, it is essential to maintain a high index of suspicion in patients with suggestive cytological features. Accurate diagnosis relies on a combination of IHC, comprehensive whole-body imaging, and targeted biopsy of the suspected primary site.

## Introduction

Metastasis to the thyroid gland is extremely uncommon. Renal cell carcinoma and cancers of the lung and breast are the most common primaries reported to metastasize to the thyroid gland. When present, secondary thyroid malignancy could mimic primary thyroid cancer, leading to diagnostic difficulties. The following case series describes three such rare presentations of secondary thyroid malignancies originating from uncommon primary cancers.

## Case presentation

### Case 1

A 55-year-old male presented to the endocrinology department with symptoms of progressive swelling in the right side of the neck and hoarseness of voice for 1 month.

### Case 2

A 54-year-old male presented to the pulmonology department with symptoms of dry cough, chest pain, and pain in the right shoulder for 10 days.

### Case 3

A 42-year-old female presented to the orthopedic oncology department with multiple swellings over the arms and back that had developed over the past 7 months.

## Diagnostic assessment

### Case 1

**Figure 1.**
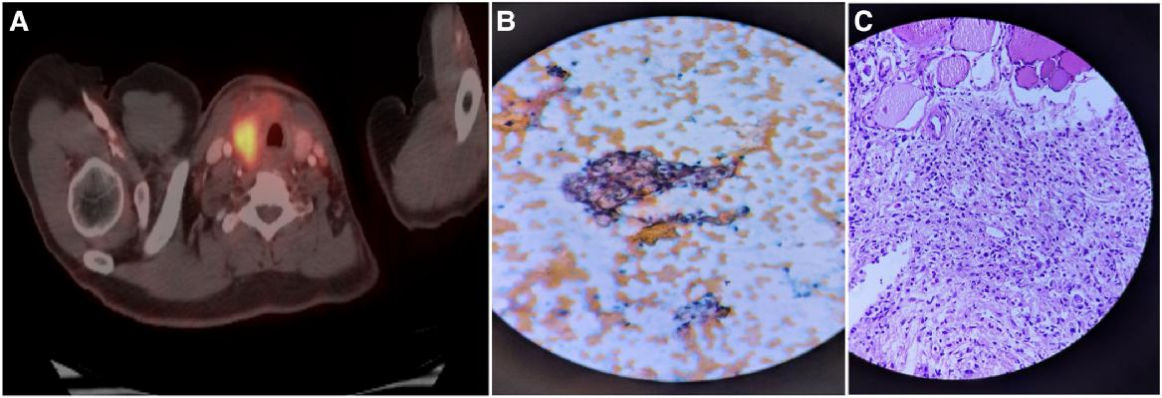
Case 1: (A) Fluorodeoxyglucose positron emission–computed tomography image showing focal fluorodeoxyglucose uptake (SUVmax 8) in the right thyroid lobe. (B) Fine-needle aspiration of thyroid nodule and cell block showing tumor cells in clusters with eccentric nuclei and abundant vacuolated cytoplasm, giving a signet ring appearance, suggesting nonthyroidal origin of the lesion. (C) Histopathological images showing the lamina propria of gastric mucosa with the tumor composed of cells with eccentric nuclei and abundant vacuolated cytoplasm (signet ring morphology) and displaying nuclear atypia with mitosis.

Examination revealed a firm, nontender, right thyroid nodule. Ultrasound (US) of the neck showed a heteroechoic solid lesion (4.3 × 2.6 cm) in the right thyroid lobe with irregular margins, categorized by the American College of Radiology Thyroid Imaging Reporting and Data System as TR4, along with suspicious right level II lymph nodes. Fluorodeoxyglucose positron emission–computed tomography (FDG PET-CT) revealed a FDG-avid heterogeneous soft tissue lesion in the right thyroid lobe and isthmus (3.3 × 2.6 × 5.7 cm, SUVmax 8) (Fig. 1A). Further evaluation of the right thyroid nodule with US-guided fine-needle aspiration cytology (FNAC) showed a poorly differentiated carcinoma with signet ring features, which was categorized as Bethesda VI (Fig. 1B). As signet ring cell adenocarcinoma most commonly originates in the stomach, an upper gastrointestinal endoscopy was performed, revealing a distal gastric ulceration with raised and irregular margins. Core biopsies obtained from the gastric ulcer (Fig. 1C) also showed poorly differentiated adenocarcinoma with signet ring morphology, confirming a primary gastric adenocarcinoma with secondary metastases to the thyroid gland. Immunohistochemistry was not performed in this case.

### Case 2

**Figure 2.**
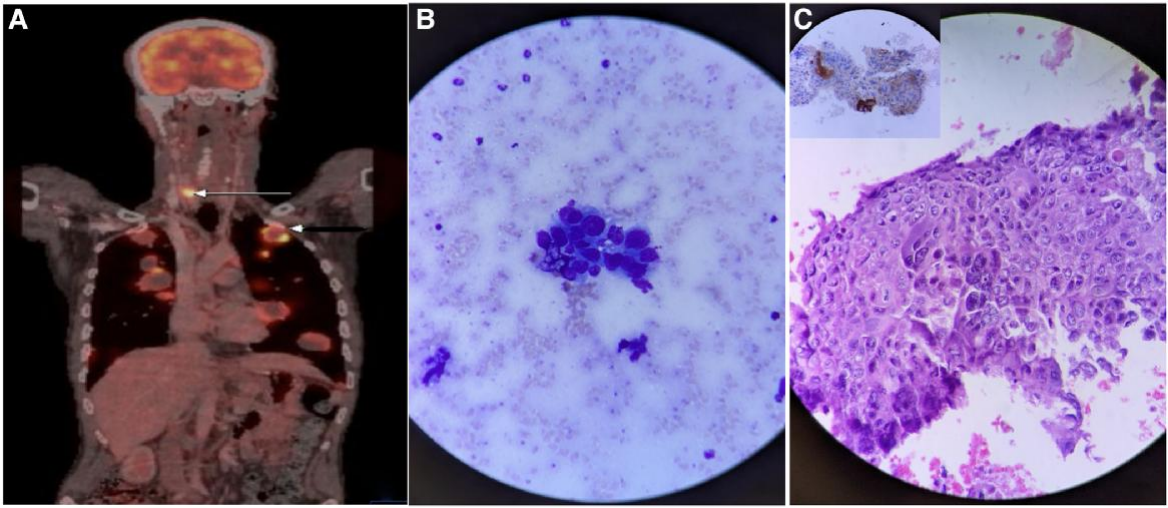
Case 2: (A) Positron emission tomography–computed tomography images showing fluorodeoxyglucose (FDG) uptake in multiple rounded heterogeneously enhancing soft tissue density lesions involving bilateral lungs (arrowhead) and a well-defined FDG-avid hypodense nodule in the right lobe of the thyroid gland (SUVmax 14.25) (arrow). (B) Fine-needle aspiration cytology smears of a thyroid nodule showing cohesive clusters of large atypical epithelial cells, displaying severe nuclear atypia. The background appears necrotic, showing some colloid with cyst macrophages. (C) Cell block section showing atypical epithelial cells with scattered multinucleated syncytiotrophoblasts, with the inset showing immunohistochemistry stain for β-human chorionic gonadotropin was positive in a few scattered trophoblasts.

A plain computed tomography scan of the thorax revealed multiple lobulated, well-circumscribed soft tissue lesions in both lungs. Subsequently, an FDG PET-CT demonstrated multiple FDG-avid, heterogeneously enhancing bilateral subpleural and parenchymal nodules. The largest lesion was in the right lung apex extending into the right upper paratracheal mediastinum (9.2 × 9.3 cm; SUVmax 9), along with another in the left lower lobe (6.4 × 7.4 cm; SUVmax 5.67) (Fig. 2A, shown by arrow). Additionally, a hypodense FDG-avid nodule was seen in the right thyroid lobe (1.9 ×1.4 cm, SUVmax 14.25) (Fig. 2A, shown by arrowhead). FNAC from the thyroid nodule showed cohesive clusters of large atypical epithelial cells, which displayed severe nuclear atypia and were categorized as Bethesda VI (Fig. 2B). Differential diagnoses included anaplastic primary thyroid carcinoma or secondary metastases from a nonthyroidal malignancy. Cell block preparation revealed large atypical epithelial cells with severe nuclear atypia with several mitotic figures and a few scattered multinucleated syncytiotrophoblasts (Fig. 2C). The IHC was positive for pan-cytokeratin and spalt-like transcription factor 4 lines with scattered cells expressing β-human chorionic gonadotropin (β-hCG), confirming choriocarcinoma (Fig. 2C inset) and negative for thyroid transcription factor 1 and paired box gene 8 (PAX8), ruling out a primary thyroid origin. Serum β-hCG level was elevated, 185 850 mIU/mL (SI units 185 850 IU/L; normal range <2 mIU/mL or <2 IU/L for males), and so was serum lactate dehydrogenase level, 694 U/L (normal range for adults 122–222 U/L in both conventional and SI units).

### Case 3

**Figure 3.**
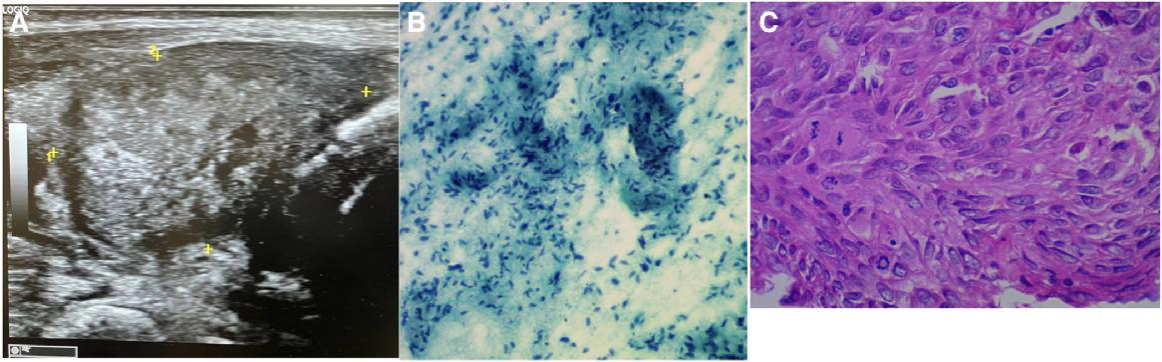
Case 3: (A) Ultrasound image of case showing American College of Radiology Thyroid Imaging Reporting and Data System Category 5 right thyroid nodule. (B) Fine-needle aspiration cytology of the thyroid nodule shows scattered spindle cells. They have oval pleomorphic nuclei with scanty cytoplasm. Few mitotic figures are seen, and no definite follicular cells are seen. (C) Biopsy from a muscle deposit showed spindle-shaped tumor cells arranged in fascicles, with moderate nuclear atypia, eosinophilic cytoplasm, and occasional giant cells, including atypical forms.

FDG PET-CT was performed, which revealed extensive visceral, nodal, and skeletal muscle metastasis along with a focal thyroid uptake. US image of the thyroid showed a right thyroid nodule (2.8 × 1.7 cm) with an American College of Radiology Thyroid Imaging Reporting and Data System of category TR5 (Fig. 3A). US-guided FNAC of the thyroid (Fig. 3B) revealed scattered spindle cells with pleomorphic nuclei and scant cytoplasm, suggesting metastatic sarcoma. No definitive follicular cells were seen. Biopsy of the right arm muscle lesion showed spindle-shaped tumor cells arranged in fascicles, with moderate nuclear atypia, eosinophilic cytoplasm, and occasional giant cells, including atypical forms (Fig. 3C). IHC on the biopsy from the arm was positive for vimentin, desmin, and smooth muscle antigen. Markers such as cluster of differentiation 10 and 34, B-cell lymphoma 2, S100 calcium-binding protein, epithelial membrane antigen, and PAX8 were negative, supporting the diagnosis of metastatic leiomyosarcoma possibly originating from the left external iliac vein.

## Treatment

### Case 1

The patient received treatment for stage IV gastric carcinoma with systemic chemotherapy, which included docetaxel and oxaliplatin.

### Case 2

The patient was initiated on chemotherapy, which included a bleomycin, etoposide, and cisplatin regimen, and tolerated it well.

### Case 3

The patient was advised of palliative chemotherapy.

## Outcome and follow-up

Cases 1 and 2 are being managed by oncology services. Case 3 was lost to follow-up.

## Discussion

Although richly vascularized, the thyroid gland is an uncommon site for clinically detectable secondary metastasis, likely due to its high oxygen and iodine content creating an inhospitable environment for tumor implantation [1, 2]. Secondary metastasis to the thyroid accounts for less than 1% of all thyroid malignancies in clinical practice, although autopsy studies show a higher incidence [1, 3]. Our case series underscores the diagnostic diversity of secondary thyroid metastasis, which often presents as a thyroid nodule in patients with either a known history of malignancy or, occasionally, as an initial clue to a nonthyroidal cancer.

In the first case, thyroid involvement was the first presentation of a primary gastric adenocarcinoma, which is rare [4]. The FDG PET-CT showed uptake in the thyroid lesion and neck nodes without any uptake in the stomach area. The presence of signet ring cells on thyroid nodule cytology raised the suspicion of gastric cancer, which led to an endoscopy that helped us establish the diagnosis. This case illustrates the value of comprehensive systemic evaluation when FNAC yields an atypical cytology.

The second case involved metastases from a germ cell tumor, identified via FNAC and IHC on a cell block. IHC is critical in such cases to differentiate metastatic disease from anaplastic thyroid carcinoma, especially when cytomorphology is ambiguous [5, 6]. The tumor cells were positive for spalt-like transcription factor 4 and β-hCG but negative for thyroid lineage markers (thyroid transcription factor 1, PAX8), which is consistent with a nonthyroidal origin. In this case, the lesion from the thyroid also offered a relatively easier site to biopsy compared to the lung in establishing the diagnosis.

The third case describes metastases from leiomyosarcoma, with FNAC showing hypercellular spindle cell clusters with marked pleomorphism. The differential diagnosis of a spindle cell cytology includes primary thyroid tumors such as medullary thyroid carcinoma, anaplastic carcinoma, and spindle epithelial tumors with thymus-like differentiation. It is nearly impossible to diagnose leiomyosarcoma through FNAC. The FDG PET-CT showed an unusual pattern of visceral and skeletal muscle spread. Core biopsy of skeletal muscle lesion with IHC staining for smooth muscle antigen, desmin, and vimentin confirmed metastatic leiomyosarcoma. Secondary metastases of soft tissue sarcoma to the thyroid are extremely uncommon, with only a few cases reported in the literature [7].

There are key cytological features distinguishing secondary thyroid metastasis from primary thyroid cancers: (a) malignant cells that appear singly or in cohesion, without a follicular pattern, (b) variable background (necrotic, bloody, or inflammatory), (c) malignant cells retain morphological features characteristic of the primary nonthyroid tumor. Therefore, although FNAC is highly sensitive and specific for primary thyroid cancers, secondary metastatic lesions often present with atypical cytologic features that require further assessment, including IHC on a cell block, IHC on a core biopsy from the thyroid lesion or from the suspected primary, and a whole-body scan [2, 8].

Metastasis to the thyroid gland remains a rare but clinically significant phenomenon that requires heightened awareness among clinicians. It could either be the presenting feature of a visceral malignancy or offer easy access to diagnose a widely spread metastatic malignancy. While incidental primary thyroid cancers are common in the evaluation of patients with nonthyroidal primaries, this case series emphasizes that a high index of suspicion is essential when evaluating thyroid nodules with atypical cytological features in this context. A multidisciplinary approach combining cytology, whole-body FDG PET-CT imaging, and IHC staining (cell block or core biopsy) is vital for definitive diagnosis. Early recognition is crucial, as the management and prognosis of secondary thyroid metastasis differ significantly from those of primary thyroid cancers.

## Learning points

- A radiologically suspicious thyroid nodule with atypical cytology suggesting a nonthyroidal origin should be carefully evaluated for possible secondary thyroid metastases.
- Although secondary thyroid metastases are typically detected as part of widespread cancer, thyroid nodules can occasionally be the presenting feature of primary malignancy elsewhere.
- IHC on a FNAC cell block or a core biopsy is helpful in confirming the diagnosis of metastasis to the thyroid and the primary tumor when used in corroboration with the FDG PET-CT whole-body scan.

## Data Availability

Restrictions apply to the availability of some or all data generated or analyzed during this study to preserve patient confidentiality or because they were used under license. The corresponding author will, on request, detail the restrictions and any conditions under which access to some data may be provided.
